# Superenhancer–transcription factor regulatory network in malignant tumors

**DOI:** 10.1515/med-2021-0326

**Published:** 2021-10-18

**Authors:** Yuan Liang, Linlin Li, Tian Xin, Binru Li, Dalin Zhang

**Affiliations:** Medical Oncology Department of Thoracic Cancer (2), Cancer Hospital of China Medical University, Liaoning Cancer Hospital & Institute, Shenyang 110042, People’s Republic of China; Department of Thyroid Surgery, The First Hospital of China Medical University, No. 155, Nanjing North Street, Shenyang 110001, Liaoning Province, People’s Republic of China

**Keywords:** malignant tumors, super enhancers, transcription factors, ChIP-seq, RNA-seq, core regulatory circuitry, superenhancer–transcription factor regulatory network

## Abstract

**Objective:**

This study aims to identify superenhancer (SE)–transcriptional factor (TF) regulatory network related to eight common malignant tumors based on ChIP-seq data modified by histone H3K27ac in the enhancer region of the SRA database.

**Methods:**

H3K27ac ChIP-seq data of eight common malignant tumor samples were downloaded from the SRA database and subjected to comparison with the human reference genome hg19. TFs regulated by SEs were screened with HOMER software. Core regulatory circuitry (CRC) in malignant tumor samples was defined through CRCmapper software and validated by RNA-seq data in TCGA. The findings were substantiated in bladder cancer cell experiments.

**Results:**

Different malignant tumors could be distinguished through the H3K27ac signal. After SE identification in eight common malignant tumor samples, 35 SE-regulated genes were defined as malignant tumor-specific. SE-regulated specific TFs effectively distinguished the types of malignant tumors. Finally, we obtained 60 CRC TFs, and SMAD3 exhibited a strong H3K27ac signal in eight common malignant tumor samples. *In vitro* experimental data verified the presence of a SE–TF regulatory network in bladder cancer, and SE–TF regulatory network enhanced the malignant phenotype of bladder cancer cells.

**Conclusion:**

The SE–TF regulatory network with SMAD3 as the core TF may participate in the carcinogenesis of malignant tumors.

## 1 Introduction

The superenhancer (SE) regulates gene expression and is characterized by high-density epigenetic modifications associated with transcription factors (TFs), and cofactors [1]. The abnormal gene transcription mediated by SEs is essential for maintaining the characteristics of tumor cells [2]. Tumor cells significantly promote the expression of various oncogenes by assembling SE, thereby enhancing their proliferation, invasion, and metastasis [3]. Therefore, the identification and analysis of core SEs and TFs in malignant tumors are of great value in tumor research [4].

Mechanisms of abnormal SE formation in malignant tumors have been demonstrated to exhibit a profound influence on both molecular pathogenesis and clinical management [5]. The transcriptional element, for example, bromodomain-containing protein 4 (BRD4) and cyclin-dependent kinase 7 (CDK7), has been reportedly the treatment target due to tumor-specific SEs [6]. Myeloid cell leukemia-1 gene (MCL1) and B-cell leukemia/lymphoma-xl (BCL-xl) are cell apoptosis regulators and express aberrantly in cancer, which plays an important role in chemoresistance [7]. By chromatin immunoprecipitation and sequencing (ChIP-seq) processing, Oldridge et al. found changed polymorphism within one SE element of LIM domain only 1 (LMO1) significantly affected neuroblastoma susceptibility by different binding of gamma-aminobutyrate (GATA) TF or regulation of LMO1 expression, which resulted in oncogenic dependency of neuroblastoma [8]. Lysine (K)-specific methyltransferase 2D (KMT2D) deficiency was reported to impair SEs to form glycolytic vulnerability in lung cancer, promote tumorigenesis in mice and upregulate protumorigenic progression, including glycolysis [9]. Oncogenic homeobox B8 (HOXB8) was driven by MYC-regulated SEs and enhanced colorectal cancer invasiveness through BTB and CNC homology 1 (BACH1) [10]. Increased expression of PPP1R15A and CDK7 is positively associated with undesirable clinical prognosis in anaplastic thyroid carcinoma. CDK7 and PPP1R15A are considered potential biomarkers and therapeutic targets for anaplastic thyroid carcinoma [11].

Although increasing reports have shown some SEs and TFs are correlated with malignancy, the common and specific SEs and TFs in various tumors, as well as complex regulatory networks, have largely been unknown. Furthermore, a majority of studies are only involved in one type of malignant tumor. Histone H3 Lys27 acetylation (H3K27ac) CHIP-seq and RNA-seq are recently hot tools for studying DNA, RNA, and protein [12,13]. Our study aims to identify and analyze core SEs and TFs in various malignant tumors such as liver cancer, lung cancer, and breast cancer based on H3K27ac ChIP-seq data in the sequence read archive (SRA) database as well as RNA-seq data in The Tumor Genome Atlas (TCGA) database.

## 2 Materials and methods

### 2.1 Data acquisition

The SRA database (https://www.ncbi.nlm.nih.gov/sra) is a database used to store the original data of second-generation sequencing. The ChIP-seq data of the differential histone modification regions of eight common malignant tumor samples were all from the SRA database. The TCGA project is a joint project initiated by the National Cancer Institute (NCI) and the National Human Genome Research Institute (NHGRI) in 2006, which included 39 types of malignant tumors investigated from the very first glioblastoma multiforme to the present, involving 29 types of malignant tumor organs, and more than 10,000 tumor samples. The RNA-seq data used in the analysis were all from the TCGA database.


**Informed consent:** Informed consent was not applicable for this study.
**Ethical approval:** Ethics committee approval was not applicable for this study.

### 2.2 Data preprocessing

Sample sequencing volume and quality were first evaluated and the Cutadapt software (https://cutadapt.readthedocs.io/en/stable/) was adopted to remove the joint and low-quality bases, followed by data quality control using the FastQC software (https://www.bioinformatics.babraham.ac.uk/projects/fastqc/). Clean reads were aligned to the human reference genome hg19 using the Bowtie2 software (ftp://igenome:G3nom3s4u@ussd-ftp.illumina.com/Homo_sapiens/UCSC/hg19/Homo_sapiens_UCSC_hg19.tar.gz). Unique alignment data were extracted from the obtained SAM files. The BAM files were deduplicated, and the data of the same cell line were merged and sorted using the Samtools [14]. With the input data as control, MACS2 software (https://pypi.org/project/MACS2/) [15] was applied to retrieve the significant H3K27ac peaks (*p* < 1 × 10^−9^ was considered significant). Significant H3K27ac peaks with a distance longer than 2.5 kb from the TSS sites were extracted as the enhancer. The obtained enhancers were extended upstream and downstream by 5 kb from the middle point of the corresponding peaks. Next, the enhancer sequence was segmented into 100 bp bin reads and aligned with the BED files converted from the BAM files generated before. The average number of peaks aligned to all the corresponding bins of an enhancer was regarded as the H3K27ac signal of the enhancer. bamCoverage in the deepTools [16] was applied to convert the processed BAM file of each sample into a corresponding Bigwig file, and the Integrated Genomics Viewer (IGV) was uploaded for visualization.

### 2.3 Histone H3K27ac modification analysis

The human reference genome hg19 was divided into small fragments with a length of 2 kb, and the number of reads of each sample mapped to each small fragment was calculated and normalized by the sequencing depth of the sample. The normalized H3K27ac signal files of the small fragments were then integrated into a matrix file according to tumor type and the correlation was calculated by an R algorithm.

### 2.4 Prediction of SEs in eight common malignant tumor samples

The SEs and TEs in all samples were calculated using the ROSE algorithm [6,17] combined with the significant H3K27ac peak found by MACS2. The SEs found in different cell lines of the same malignant tumor were merged through the merge module of Bedtools software [18], and then the frequency of occurrence of each merged SE in different cell lines was calculated. In each malignant tumor, the SEs appearing in at least two cell lines were selected for subsequent analysis.

### 2.5 Differential expression analysis of SEs and TEs

The SEs that appeared in at least two cell lines were annotated with HOMER software [19]. Subsequently, the SE-regulated target genes in each malignant tumor sample were extracted for functional enrichment analysis with DAVID software [20,21]. These genes were merged using the merge module of the Bedtools software, and the frequency of all SEs presented in eight common malignant tumors was calculated. The number of different SE-regulated target genes in eight common malignant tumor samples was counted and the conservative SE-regulated target gene and the malignant tumor-specific SE-regulated target gene were defined in sequence. Afterward, a malignant tumor conservative SE-regulated target gene and a malignant tumor-specific SE-regulated target gene were selected and displayed (H3K27ac signal) using IGV. Finally, expression profile data in the TCGA database was used for verification.

### 2.6 Identification of conservative and malignant tumor-specific TFs

Single significant H3K27ac peaks included in the SEs that appeared more than twice in eight common malignant tumors were first extracted. The nucleosome-free regions (NFRs) were then extracted from the bed file converted from the sorted BAM file with HOMER. The single significant H3K27ac peaks were overlapped with the extracted NFRs to identify the NFRs located on the SEs, and the results were saved as NFR bed files. The bed files were used as input to find the TFs regulated by SEs in corresponding malignant tumor samples with HOMER, and the *p*-value and motif graphs of the corresponding TFs were generated. The frequency of occurrence of TFs binding to SEs in eight common malignant tumor samples was counted, and conservative and malignant tumor-specific TFs were defined according to the significance of all the TFs in each malignant tumor sample (the significance was indicated by the *p*-value). The expression of all TFs in each malignant tumor sample was calculated through the expression profile in TCGA (the average value of FPKM in all samples obtained) for verification of the conservative and malignant tumor-specific TFs. Finally, a conservative TF gene and a malignant tumor-specific TF were selected and their expression in each malignant tumor sample was calculated and shown by the *Beeswarm* package (https://github.com/aroneklund/beeswarm).

### 2.7 Screening of the SE–TE regulatory network in eight common malignant tumors

Core regulatory circuitry (CRC), namely the SE–TE regulatory network, mainly refers to the regulatory loop composed of SEs and core TFs in cells [22]. Generally, the expression of a core TF gene was not only regulated by the corresponding SEs but also regulated by binding of the SEs with the TF itself. CRCmapper software [22] was applied to define the core TF regulatory circuits in each malignant tumor sample. The TFs with the highest score in the SE–TF regulatory network in each malignant tumor sample were counted and collated according to the frequency of their appearance in each malignant tumor sample. For further validation, the expression of all TF coding genes was displayed according to the tumor expression profile data in the TCGA database, and then a CRC TF that appeared several times in malignant tumor core TFs, as well as a specific TF that only appeared in one specific malignant tumor sample, was selected to separately display the H3K27ac signal of the SEs near the two loci by IGV.

### 2.8 Cell culture and transfection

Human gastric malignant tumor cell line MKN45, human renal malignant tumor cell line 786-M1A, human esophageal squamous cell line KYSE140, human colorectal malignant tumor cell line HCT116, human bladder malignant tumor cell line T24, and human breast malignant tumor cell line 76NF2V, small cell lung malignant tumor cell line COR-L311, and human liver malignant tumor cell line HepG2 were purchased from American Type Culture Collection (ATCC; Manassas, VA, USA). MKN45, 786-M1A, T24, and COR-L311 cells were cultured with the RPMI-1640 medium (Gibco BRL, Grand Island, NY, USA) containing 10% fetal bovine serum (FBS; Gibco), 10 μg/mL streptomycin, and 100 U/mL penicillin, while HCT116, 76NF2V, and HepG2 cells were cultured in Dulbecco’s modified Eagle’s medium (DMEM; Sigma-Aldrich Chemical Company, St Louis, MO, USA) containing 10% FBS (Gibco), 10 μg/mL streptomycin, and 100 U/mL penicillin. KYSE140 cells were cultured with minimum essential medium (MEM) supplemented with 10% FBS (Gibco), 10 μg/mL streptomycin, and 100 U/mL penicillin. The aforementioned cells were incubated in a 5% CO_2_ incubator (Thermo Fisher Scientific Inc., Waltham, MA, USA) at 37°C.

T24 cells in the logarithmic phase were trypsinized, seeded into a 6-well plate at a density of 1 × 10^5^ cells per well, and cultured for 24 h. Upon reaching about 75% confluence, the cells were transfected using the Lipofectamine 2000 reagent (Invitrogen Inc., Carlsbad, CA, USA) with short hairpin RNA plasmids targeting NC (sh-NC), SMAD3 (sh-SMAD3), ETS1 (sh-ETS1), and HOXB2 (sh-HOXB2), and overexpression plasmids of NC (pcDNA3.1), SMAD3 (pcDNA3.1-SMAD3), ETS1 (pcDNA3.1-ETS1), and HOXB2 (pcDNA3.1-HOXB2), as well as sh-NC + pcDNA3.1, sh-SMAD3 + pcDNA3.1, sh-SMAD3 + pcDNA3.1-ETS1, sh-SMAD3 + pcDNA3.1-HOXB2, and sh-SMAD3 + pcDNA3.1-ETS1 + pcDNA3.1-HOXB2 plasmids. After 48 h of transfection, subsequent experiments were carried out, and each experiment was repeated 3 times. The concentration of plasmids used was 50 ng/mL. shRNA or overexpression plasmids were provided by Shanghai GenePharma Co., Ltd (Shanghai, China).

### 2.9 Chromatin immunoprecipitation (ChIP)

ChIP kit (Thermo Fisher Scientific) was used for this experiment. The treated cells were fixed with 1% formaldehyde and sonicated to trigger DNA strand breaks. The complex was immunoprecipitated by incubation with rabbit antibodies against IgG (1:100, ab6757, Abcam Inc., Cambridge, UK), H3K27ac (1:50, ab4727, Abcam), SMAD3 (1:50, ab208182, Abcam), ETS1 (1:50, ab124282, Abcam), and HOXB2 (1:50, ab220390, Abcam). Next, the complex was filtered from the DNA fragments through protein G–Sepharose beads. Cross-linking of the DNA complex was relieved and DNA strands were purified. Reverse transcription-quantitative polymerase chain reaction (RT-qPCR) was performed to quantify ChIP products. The primer sequences are shown in Table S1.

### 2.10 RNA isolation and quantitation

Total RNA was extracted from cells using the TRIzol reagent (16096020, Thermo Fisher Scientific). For mRNA analysis, the extracted RNA was reversely transcribed into cDNA using a reverse transcription kit (RR047A, Takara, Tokyo, Japan). RT-qPCR was conducted using an SYBR® Premix Ex TaqTM II kit (DRR081, Takara) on an ABI 7500 instrument (Applied Biosystems, Foster City, CA, USA), with three repeated wells for each sample. GAPDH served as the internal reference, and the fold changes were calculated using the method of 2^−ΔΔCt^. The primer sequences are shown in Table S2.

### 2.11 Western blot analysis

Total protein was extracted from cells with the radio-immunoprecipitation assay (RIPA) lysis buffer (C0481, Sigma-Aldrich) containing 1% protease inhibitor and 1% phosphorylase inhibitor (Shanghai Beyotime Biotechnology Co., Ltd., Shanghai, China). The protein concentration was then determined with a bicinchoninic acid kit (23227, Thermo Fisher Scientific). The protein was quantified in 5× loading buffer (P0015, Beyotime), separated by sodium dodecyl sulfate-polyacrylamide gel electrophoresis, and transferred onto a polyvinylidene fluoride membrane (Millipore, Billerica, MA, USA). Next, the membrane was treated with 5% bovine serum albumin (BSA) at room temperature for 1 h and incubated overnight at 4°C with primary rabbit antibodies against SMAD3 (1:1,000, ab208182, Abcam), ETS1 (1:2,000, ab124282, Abcam), HOXB2 (1:5,000, ab220390, Abcam), and β-actin (1:5,000, ab8227, Abcam). The following day, the membrane was incubated with horseradish peroxidase-labeled secondary antibody goat anti-rabbit IgG (1: 20,000, ab205718, Abcam) at room temperature for 1.5 h. Afterward, the membrane was developed using the developing solution (NCI4106, Pierce, Rockford, IL, USA), after which the protein bands were quantified by ImageJ 1.48 u software (Bio-Rad, Inc., Hercules, CA, USA). The ratio of the gray value of the target band to that of β-actin was representative of the relative protein expression.

### 2.12 Transwell assay

Transwell chambers (8 μm pore size; Corning Incorporated, Corning, NY, USA) in 24-well plates were used for *in vitro* cell migration and invasion detection. In brief, 600 μL of 20% FBS-containing RPMI-1640 medium was preadded to the 8 μm pore-size Matrigel-coated Transwell chambers and the Matrigel-free Transwell chambers and equilibrated at 37°C for 1 h. T24 cells transfected for 48 h were resuspended in RPMI-1640 medium containing 10% FBS, and 100 μL of the cell suspension containing 1 × 10^9^ cells/L was added to the upper chamber, and cultured at 37°C with 5% CO_2_ for 24 h. The Transwell chamber was removed and the cells in the inner layer of the Transwell chamber were wiped with a cotton swab. After washing with PBS, cells were fixed 4% methanol, and stained with 0.1% crystal violet before counting; they were photographed under an inverted microscope (Olympus IX73, Olympus Optical Co., Ltd, Tokyo, Japan) in five randomly selected fields of view, with three replicates for each specimen. The differences between the groups were analyzed and the histogram of migration and invasion was plotted.

### 2.13 Cell count kit-8 (CCK-8) assay

T24 cell proliferation was evaluated using a CCK-8 kit (K1018, Apexbio, USA). Cells (1 × 10^4^ cells per well, 100 μL/well) were plated in a 96-well plate, and 10 μL of CCK-8 solution was added at each time point (0, 24, 48, 72, and 96 h) to incubate the cells at 37°C for 2 h. Next, the optical density (OD) value was measured at 450 nm with a microplate reader, and the obtained data were displayed in curve graphs.

### 2.14 Statistical analysis

All data were analyzed using SPSS 21.0 statistical software (IBM Corp. Armonk, NY, USA). The measurement data were described as mean ± standard deviation. Data obeying normal distribution and homogeneity of variance between two groups were compared by unpaired *t*-test. Differences among multiple groups were statistically analyzed using one-way analysis of variance (ANOVA) or repeated measures ANOVA, followed by Tukey’s *post hoc* tests with corrections for multiple comparisons. A value of *p* < 0.05 was statistically significant.

## 3 Results

### 3.1 Data information of cell lines in eight common malignant tumor samples in the SRA database

ChIP-seq data of differential histone modification regions in eight common malignant tumor cell lines were downloaded from the SRA database. All cell lines did not receive special treatment, and the data of different cell lines for each malignant tumor were merged and 71 types of cell lines were obtained. The data information of eight common malignant tumor cell lines in the SRA database is shown in Table S3.

### 3.2 Difference in the H3K27ac signal intensity in the enhancer region of eight common malignant tumor cell lines

**Figure 1:**
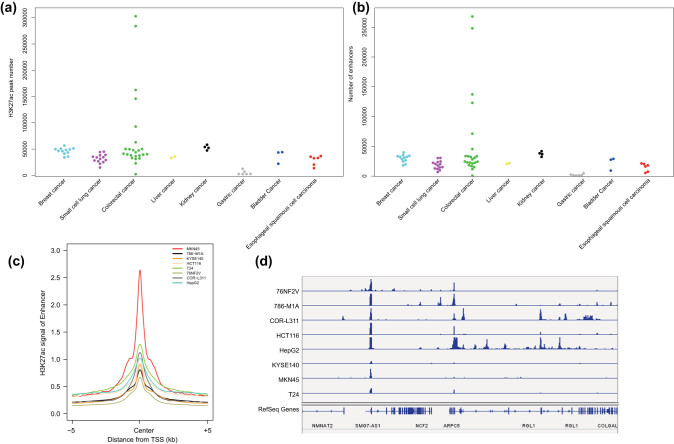
H3K27ac signal intensity on the enhancers of the eight common malignant tumor cell lines. (a) The number of significant H3K27ac signal peaks in eight common malignant tumor cell lines. (b) The number of enhancers in eight common malignant tumor cell lines. (c) H3K27ac signal intensity on the enhancers of the eight common malignant tumor cell lines. (d) H3K27ac signal intensity in the NCF2 gene region in the eight common malignant tumor cell lines.

Analysis of the histone H3K27ac modification changes in the enhancer region of each malignant tumor cell line by the MACS2 software indicated a large number of significant peaks in each malignant tumor cell line (Figure 1a). In addition, the Python script results showed the presence of a large number of enhancers in each malignant tumor cell line, which was consistent with the significant H3K27ac peak trend (Figure 1b). To ensure that the prediction of the enhancers was accurate, we selected a malignant tumor cell line for each malignant tumor (gastric malignant tumor: MKN45; renal malignant tumor: 786-M1A; esophageal squamous cell carcinoma: KYSE140; colorectal malignant tumor: HCT116; bladder malignant tumor: T24; breast malignant tumor: 76NF2V; small cell lung malignant tumor: COR-L311; hepatocellular carcinoma: HepG2) and displayed the H3K27ac signals in the predicted enhancer regions. A strong signal peak of H3K27ac was observed in the middle of the enhancer in each malignant tumor sample (Figure 1c). Furthermore, we displayed the H3K27ac signals on the neutrophil cytosolic factor 2 (NCF2) gene locus by IGV in the eight selected malignant tumor cell lines, as representative; and we found that H3K27ac signals were highly enriched in the enhancer regions (Figure 1d).

### 3.3 The type of H3K27ac modification signal is different for each malignant tumor

**Figure 2:**
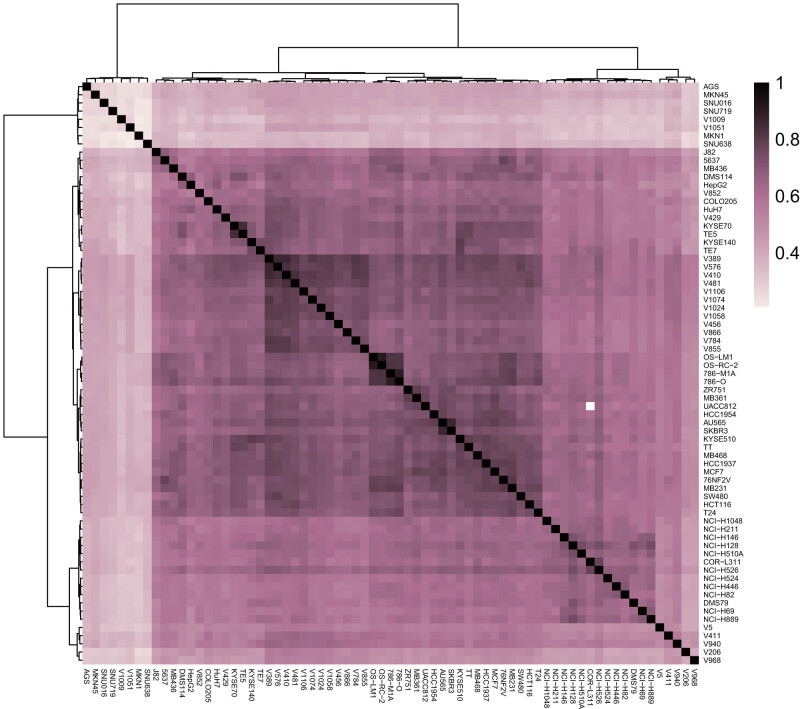
A heat map of the correlation analysis of the H3K27ac signals in different malignant tumor cell lines.

Correlation analysis of H3K27ac signals among malignant tumor samples revealed the H3K27ac signals of the same type of malignant tumor cell lines could be clustered together, while the H3K27ac signals of different types of malignant tumor cell lines failed to be clustered. This finding indicated that the H3K27ac modification signal type of each malignant tumor was different and different malignant tumors could be thus distinguished by the H3K27ac signal type (Figure 2).

### 3.4 Screening of the number of SEs in eight common malignant tumor samples

**Figure 3:**
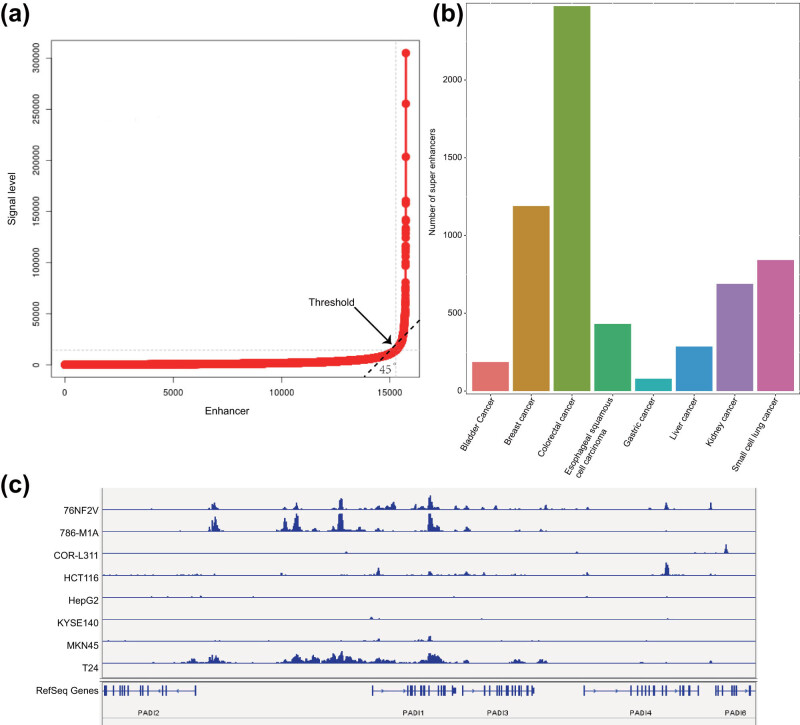
Prediction of SEs in eight common malignant tumor cell lines. (a) Ranking result of the H3K27ac signal intensity of the stitched enhancers and the remaining single enhancers. The arrow indicates the threshold point. (b) The number of SEs that occurred in eight common malignant tumor cell lines. The abscissa represents the type of malignant tumor, and the ordinate represents SEs appearing at least twice in each malignant tumor cell line. (c) The difference in the signal intensity of H3K27ac in the enhancer region of the PADI1 gene in eight common malignant tumor samples.

SEs are a large cluster of transcriptionally active enhancers that drive the expression of genes that control cell identity [23]. To identify the SEs in all the eight common malignant tumors, we applied a method consisting of three steps. The first step was to determine the active enhancer sites, which are described in Figure 1. The next step was to stitch the enhancers we obtained by their distribution and distance between each other. Within the genome range, if the distance between two enhancer annotations were within 12.5 kb, then they were merged into a single entity called the stitched enhancer. Finally, the threshold between the SEs and the TEs was determined. We sorted the stitched enhancers and the remaining single enhancers according to the intensity of the H3K27ac signal level (from low to high) and drafted a graph of the ranking result. On this graph of H3K27ac signal density vs density ranking, we identified the tangent point by a tangent line with a slope of 1, which was considered as the threshold. That is, the points with higher density (to the right and above the tangent point) were SEs, while the rest were TEs (Figure 3a). The significant H3K27ac peak found by the ROSE algorithm combined with the screening results of MACS2 software was used to calculate the SEs and TEs in all samples. The SEs found in different cell lines of the same type of malignant tumor were merged using the merge module of Bedtools software, and the SEs that appeared in at least 2 cell lines in the eight common malignant tumor samples were calculated. The results indicated that eight common malignant tumor samples had a large number of SEs, and colorectal malignant tumor samples had the largest number (2500) (Figure 3b). To further confirm that our prediction of SEs was correct, we selected the PADI1 gene, which was reported to be regulated by an adjacent estrogen receptor alpha-dependent SE [24] and visualized by IGV. The results showed that, except for the COR-L311, HepG2, and KYSE140 cell lines, other cell lines all had strong PADI1 gene enhancer region H3K27ac signal expression, suggesting SE expression (Figure 3c).

### 3.5 Analysis of conservative and malignant tumor-specific target genes regulated by SEs in eight common malignant tumor samples

**Figure 4:**
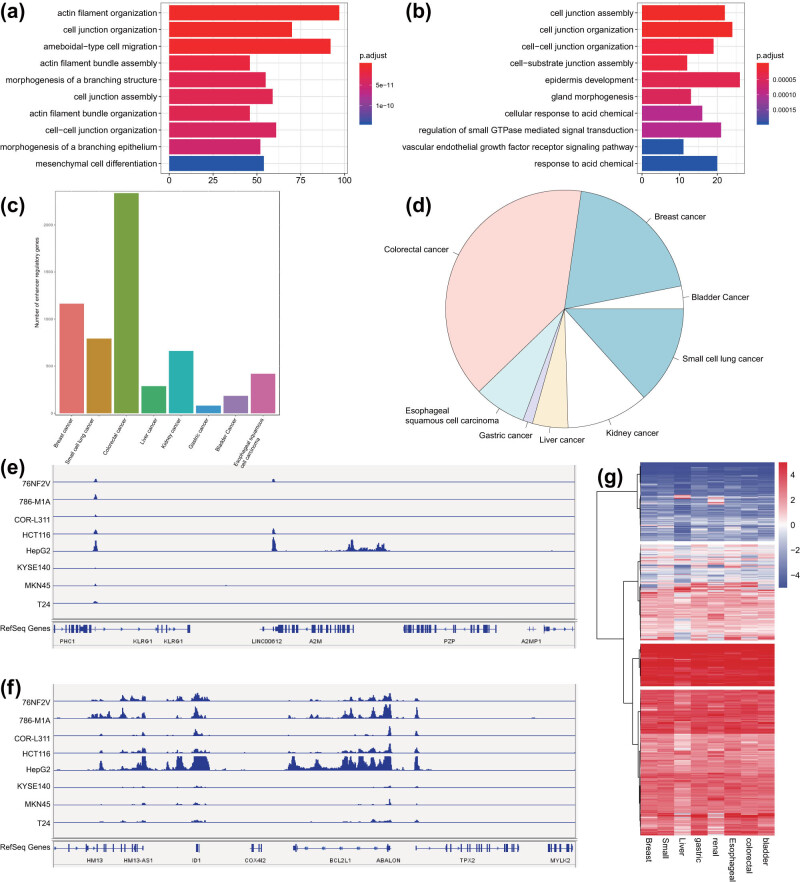
Analysis of conservative and malignant tumor-specific SEs in eight common malignant tumor cell lines. (a) GO functional enrichment analysis of SE-regulated genes in colorectal cancer samples. The ordinate represents the enriched pathway, and the abscissa represents the number of genes enriched in the pathway. The color represents the significance, with deep red indicating more significance. (b) GO functional enrichment analysis of SE-regulated genes in bladder cancer samples. The ordinate shows the enriched pathway, and the abscissa indicates the number of genes enriched in the pathway. The color represents the significance of the pathway, with deep red indicating more significance. (c) The number of SE-regulated genes in eight common malignant tumor cell lines. (d) The frequency of SE-regulated target genes in eight common malignant tumor cell lines following consolidation. (e) H3K27ac signal distribution on the specific SE-regulated target gene *A2M* in the eight common malignant tumor cell lines. (f) H3K27ac signal distribution on the conservative SE-regulated target gene *ABALON* in the eight common malignant tumor cell lines. (g) Expression of SE-regulated target genes in the eight common malignant tumor cell lines in TCGA database.

Functional enrichment analysis of the SE-regulated genes in malignant tumor samples through DAVID software revealed that these genes were significantly enriched in pathways including cell junction organization, cell junction assembly, and cell–cell junction organization (Figure 4a and b). We collated all SE-regulated genes in eight common malignant tumor samples and obtained 5,923 target genes (Figure 4c and d). As shown in Table S4, there were 2154 SE-regulated genes (conservative target genes) occurring more than 6 times in eight common malignant tumor samples, while 35 genes (specific target genes) occurring only once. We selected the malignant tumor-specific SE-regulated gene A2M and the conservative SE-regulated gene ABALON as representatives and displayed the H3K27ac signal distribution on the two loci by IGV in the eight common malignant tumor cell lines. The results showed that H3K27ac signal peaks on A2M were only observed in a few malignant tumor cell lines (Figure 4e), while the peaks on ABALON was clearly observed in eight common malignant tumor cell lines (Figure 4f). Analysis of the SE-regulated target gene expression data in the eight common malignant tumor samples from the TCGA database revealed that the results were consistent with the H3K27ac ChIP-seq results (Figure 4g).

### 3.6 Identification of malignant tumor-specific and conservative TFs regulated by SEs in eight common malignant tumor samples

**Figure 5:**
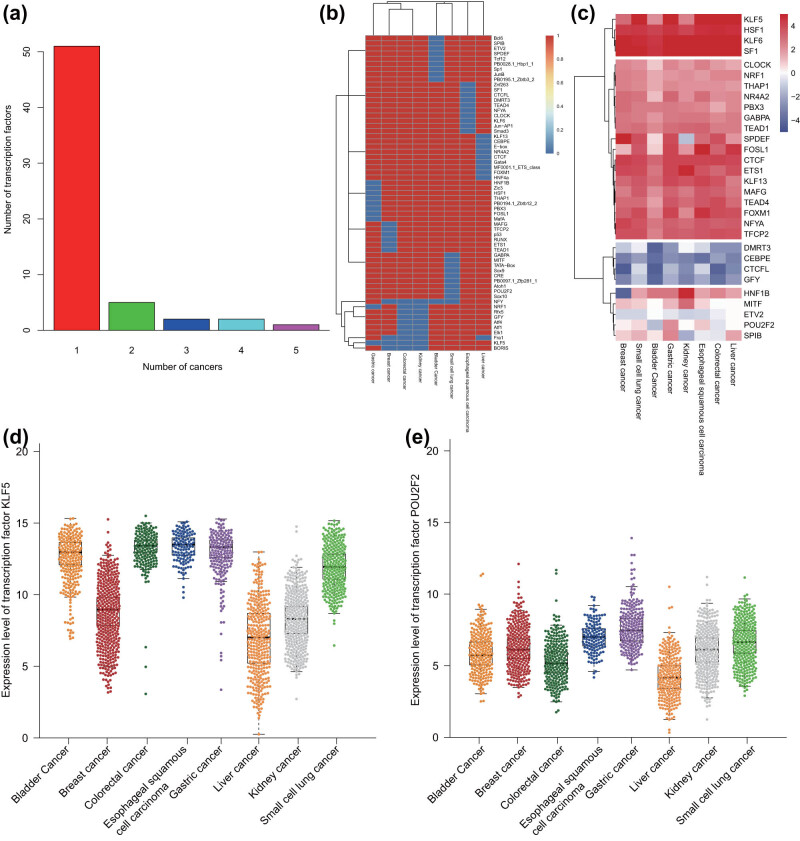
Identification of SE-related malignant tumor-specific and -conservative TFs in eight common malignant tumor samples. (a) The frequency and number of TFs regulated by SEs in eight common malignant tumor samples. (b) A heat map of all the identified TFs in each malignant tumor sample. (c) The expression of all the identified TFs in TCGA RNA-seq data of each malignant tumor sample. The expression was evaluated by taking the average value of FPKM of all samples in TCGA RNA-seq data. (d) The expression of the conservative TF KLF5 in TCGA RNA-seq data of each malignant tumor sample. (e) The expression of the malignant tumor-specific TF POU2F2 in TCGA RNA-seq data of each malignant tumor sample.

The TFs regulated by SEs in each malignant tumor sample were identified using HOMER software, and the top 10 TFs with the smallest *p*-value were screened. The results displayed that some TFs existed in multiple malignant tumor samples, and some TFs only in specific malignant tumor samples (Figure 5a). The differential expression of all TFs in each malignant tumor sample is shown in Figure 5b. The TF occurring more than 4 times was defined as a conservative TF and that occurring only once was defined as a specific TF. Based on TCGA RNA-seq data of eight common malignant tumor samples, we calculated the expression of all the identified TFs in each malignant tumor type (Figure 5c). Finally, we selected a conservative TF kruppel-like factor 5 (KLF5) and malignant tumor-specific TF POU class 2 homeobox 2 (POU2F2) and analyzed their expression in each type of malignant tumor samples. The data exhibited that the expression of KLF5 was almost similar in 4–5 malignant tumor samples (Figure 5d), while that of POU2F2 was upregulated only in small cell lung cancer samples (Figure 5e). These results indicated that the specific TFs regulated by SEs could distinguish the malignant tumor types effectively.

### 3.7 Screening of the SE–TF regulatory network in eight common malignant tumor cell lines

**Figure 6:**
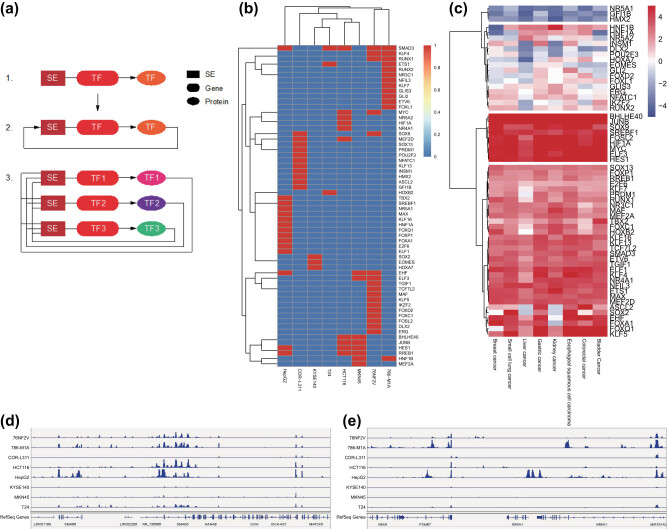
Screening of the SE–TF regulatory network in eight common malignant tumor cell lines. (a) Schematic diagram of SE–TF network. (b) A heat map of the top 10 core TFs in each malignant tumor sample. (c) A heat map of the expression of core TFs in TCGA RNA-seq data. (d) H3K27ac signal intensity of SMAD3 in eight common malignant tumor cell lines. (e) H3K27ac signal intensity of NR5A1 in eight common malignant tumor cell lines.

The screening of the SE–TF regulatory network was divided into three steps: the first step was to find all the TFs regulated by the SEs; the second step was to find the TFs that could bind to the SEs to regulate the expression of their own genes from the TFs regulated by SEs, which is the so-called autoregulated; the third step was to merge all autoregulated TFs. These TFs could regulate the expression of each other as well as themselves, and they together formed a SE–TF regulatory network (Figure 6a).

Prediction results of the core TFs in eight common malignant tumor cell lines by CRCmapper software are shown in Table S5. The core TFs with the highest scores in each malignant tumor sample were merged, with a total of 60 core TFs obtained (Figure 6b). To verify the prediction, we analyzed TCGA RNA-seq data of eight common malignant tumor samples and found consistent results with those of CRCmapper software (Figure 6c). We further validated the results by selecting TFs SMAD3 and NR5A1 and employed IGV to analyze the H3K27ac signal in the gene enhancer region. The results showed that SMAD3 had strong H3K27ac signals in eight common malignant tumor samples, while the NR5A1 only had H3K27ac signals in liver cancer samples (Figure 6d and e). The above results indicated that the SE–TF regulatory network with the TF SMAD3 as the core may be involved in the occurrence and development of a variety of malignant tumors.

### 3.8 Validation of the identified SE–TF regulatory network in bladder cancer cells

**Figure 7:**
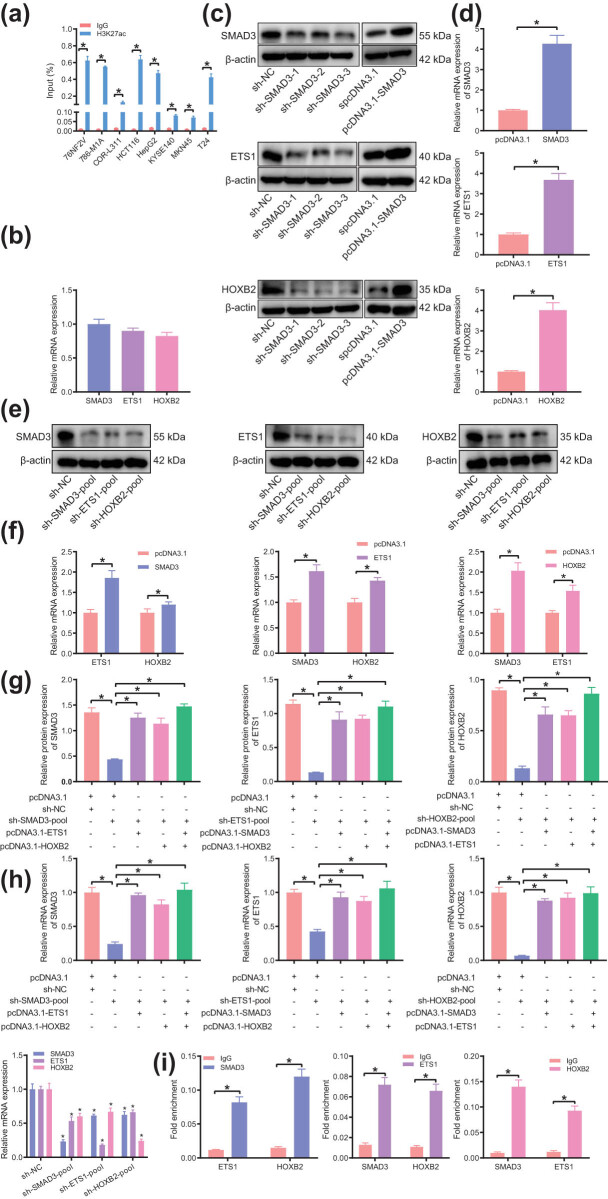
The presence of SE–TF regulatory network consisting of SMAD3, ETS1, and HOXB2 in T24 cells. (a) H3K27ac intensity of SMAD3 in eight common malignant tumor cell lines analyzed by ChIP-PCR. (b) Expression of core TFs of bladder cancer (SMAD3, ETS1, and HOXB2) in T24 cells determined by RT-qPCR. (c) Silencing and overexpression efficiency of SMAD3, ETS1, and HOXB2 in T24 cells determined by western blot analysis. (d) Overexpression efficiency of SMAD3, ETS1, and HOXB2 in T24 cells determined by RT-qPCR. (e) Expression of other two TFs in T24 cells following silencing of any bladder cancer TFs determined by western blot analysis. (f) Expression of other two TFs in T24 cells following overexpression of any bladder cancer TFs determined by RT-qPCR. (g) Expression of other TFs in T24 cells following intervention with two types of bladder cancer TFs determined by western blot analysis. (h) Expression of other TFs in T24 cells following intervention with two types of bladder cancer TFs determined by RT-qPCR. (i) The binding of each TF to the promoter regions of the other two TFs analyzed by ChIP-PCR. *p < 0.05. The experiment was conducted three times independently.

Next, we aimed to verify the H3K27ac signal of the TF SMAD3 in tumor samples by culturing eight kinds of malignant tumor cell lines. The results of ChIP-PCR showed SMAD3 had a strong H3K27ac signal in eight common malignant tumors (Figure 7a), suggesting that the SE–TF regulatory network with SMAD3 as the core may be involved in the occurrence and development of a variety of malignant tumors.

Further, we took bladder cancer as an example for verification. The core TFs of bladder cancer were SMAD3, ETS1, and HOXB2. The results of RT-qPCR showed (Figure 7b) these TFs were expressed at an average level in the bladder cancer cell line T24. T24 cells were transfected with shRNA and overexpression plasmids targeting these TFs, the efficiency of which was confirmed by RT-qPCR and western blot analysis (Figure 7c and d). In order to achieve the best silencing efficiency, the siRNA-pool was transfected into T24 cells where the expression of TFs was determined by RT-qPCR and western blot analysis. The results presented that silencing the expression of any TFs would result in a decrease in the expression of other TFs, and meanwhile, overexpression of any TFs would also lead to an increase of other TFs (Figure 7e and f). In addition, silencing any TFs and overexpressing other TFs simultaneously in T24 cells could partially reverse the above results (Figure 7g and h). These results indicated that SMAD3, ETS1, and HOXB2 in bladder cancer interacted with each other to construct a SE–TF regulatory network.

For further verification of the direct transcriptional regulation between these TFs, we used ChIP-PCR to detect the binding of each TF to the promoter regions of the other two TFs. The results displayed that each TF could bind to the promoter regions other than itself (Figure 7i), indicating that all TFs were connected to each other to form a network. The above results demonstrated the presence of a SE–TF regulatory network in bladder cancer.

### 3.9 SE–TF regulatory network enhances the malignant phenotype of bladder cancer cells

**Figure 8:**
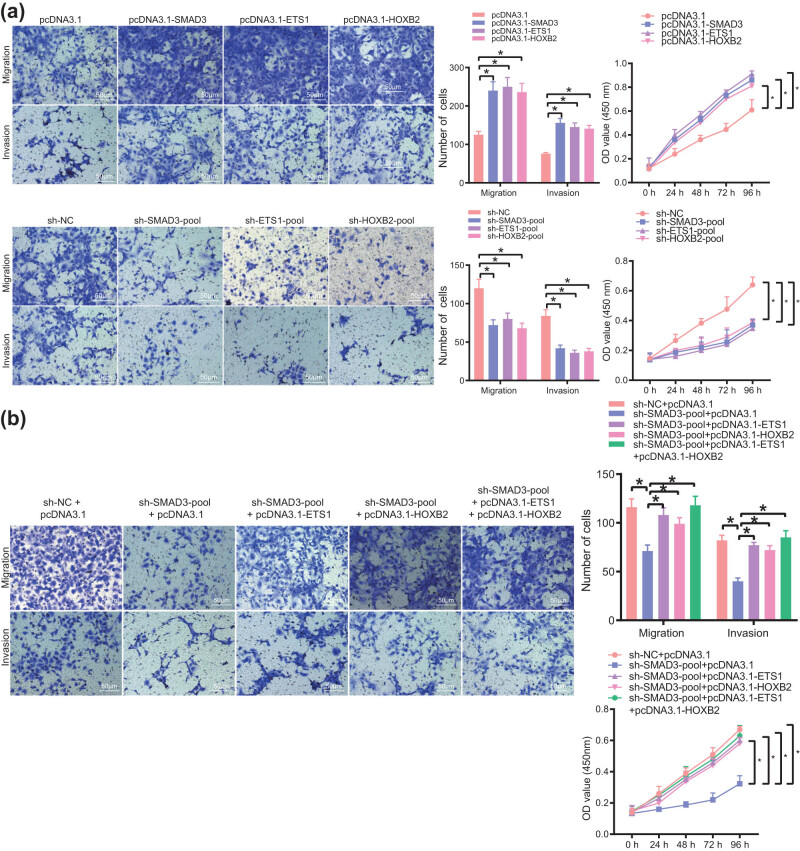
The SE–TF regulatory network consisting of SMAD3, ETS1, and HOXB2 promotes the malignant phenotype of bladder cancer cells. (a) Migration, invasion, and proliferation of T24 cells following overexpression or silencing of SMAD3, ETS1, and HOXB2 measured by Transwell and CCK-8 assays. (b) Migration, invasion, and proliferation of T24 cells following transfection with sh-SMAD3, sh-SMAD3 + pcDNA3.1-ETS1, sh-SMAD3 + pcDNA3.1-HOXB2, or sh-SMAD3 + pcDNA3.1-ETS1 + pcDNA3.1-HOXB2 measured by Transwell and CCK-8 assays. *p < 0.05. The experiment was conducted three times independently.

Finally, we attempted to elucidate the effect of the SE–TF regulatory network on the function of T24 cells. The results of Transwell and CCK-8 assays showed that overexpression of TFs stimulated the migration, invasion of T24 cells, accompanied by enhanced proliferation while silencing of TFs resulted in opposite results (Figure 8a). Furthermore, weakening of the malignant phenotype of T24 cells caused by silencing of SMAD3 could be partially reversed by overexpression of other one or two TFs (Figure 8b). The aforementioned findings indicated the promoting effect of the SE–TF regulatory network on the malignant phenotype of bladder cancer cells.

## 4 Discussion

SEs and TFs are associated with many genes involved in cancer pathogenesis [5,25]. Primary oncogene promoters of tumor cells are controlled by SE, rendering selective activation of transcription [26]. Mechanisms of abnormal SE formation in malignant tumors have been demonstrated to exhibit profound influence on both molecular pathogenesis and clinical management [5]. Our study is to identify and validate core SEs and TFs in eight common malignant tumors based on H3K27ac ChIP-seq in the SRA database as well as RNA-seq data in the TCGA database, followed by verifications in bladder cancer cell experiments.

First, we obtained H3K27ac and Input ChIP-seq sequencing data of eight common malignant tumor cell lines including gastric cancer through the SRA database and downloaded FPKM data of all RNA-seq from the TCGA database for verification. Similarly, other research works also downloaded H3K27ac and Input ChIP-seq sequencing data of tumor cell lines from the SRA database [27,28,29]. Unique to our result, FPKM data of all RNA-seq were downloaded from the TCGA database for verification; ChIP-seq sequencing data were not specially processed and from the same malignant tumor cell lines before merging. This study targets eight common malignant tumors, whereas the former reports only refer to a kind of malignant tumor [3,30]. Then, MACS2 software was used to find the H3K27ac peak in each malignant tumor cell line. Taking the significant H3K27ac peak at more than 2.5 kb from the TSS site as the enhancer, we found that abundant enhancers existed in each malignant tumor cell line, which was consistent with the tendency of the significant H3K27ac peak. The NCF2 gene was selected and IGV was used to show the H3K27ac signal on the enhancer of eight common malignant tumors. Generally, Bowtie2 is used for comparison in the preliminary processing of ChIP-seq sequencing data, and the only comparison data are obtained by Samtools to deduplicate [31,32,33]. Differently, this study merged data from the same tumor cell line and identified the significant H3K27ac peak through the MACS2 software. The significant H3K27ac peak at more than 2.5 kb from the TSS site was used as the enhancer. Then the significant H3K27ac peak was compared with the identified enhancer, and finally, all data analyzed were displayed as a whole. Next, we found that H3K27ac signals of same malignant tumor cell line could be clustered together by analyzing the correlation of H3K27ac signals, indicating that H3K27ac pattern is different and the H3K27ac signal can distinguish malignant tumors. We used the ROSE algorithm to predict SEs. A single enhancer entity within the range of 12.5 kb was merged and SEs that occurred in at least two cell lines were calculated so that the range was diminished and the stability of the prediction results was improved.

In addition, we examined all SE-regulated genes in eight common malignant tumors. Thirty-five genes that occurred more than six times in all malignant tumors were defined as conservative SE-regulated genes, and 2,154 genes were specific for each malignant tumor sample. The data analyzed in this study were only for SEs that occurred in at least two cell lines, and the specific and conservative SEs were defined differently. The defined SEs were verified by IGV, and the SE-regulated genes in all malignant tumors were obtained from the TCGA database. Finally, the expression data in eight common malignant tumors were compared with the analysis results of H3K27ac ChIP-seq data. Generally, the core TFs that bound to SEs in each malignant tumor sample were found with HOMER software [34,35,36]. Importantly, the TFs that appeared more than four times in malignant tumors were defined as malignant tumor-conservative TFs, while TFs that appeared only once were defined as malignant tumor-specific TFs. Based on TCGA RNA-seq data, we calculated the expression of all TFs in each type of malignant tumor, which can be distinguished according to the defined specific and conservative TFs. Conservative TF KLF5 and specific TF POU2F2 were chosen to exhibit the expression of all malignant tumors through a beeswarm package, which also could distinguish malignant tumor-specific TFs from conservative TFs. Finally, CRC TFs were predicted in eight common malignant tumor cell lines through the CRCmapper software, which is the same as other reports [37,38]. This study combined with the expression profile data of patients in the TCGA database, and also showed some of the identified CRC TFs near SE H3K27ac signal through the IGV. Furthermore, the *in vitro* experimental data demonstrated the presence of a SE–TF regulatory network in bladder cancer, and the SE–TF regulatory network enhanced the malignant phenotype of bladder cancer cells.

In conclusion, we integrated the H3K27ac ChIP-seq of eight common malignant tumor cell lines and RNA-seq from cancer patients in the TCGA database and identified core SEs and TFs. In all malignant tumor samples, there were 35 SE-regulated genes that occurred more than six times, while 2,154 SE-regulated genes occurred only once, which were defined as conservative SE-regulated genes and specific SE-regulated genes, respectively. We found the core TFs bound to SEs in each malignant tumor sample with HOMER. The TFs that appeared more than four times in tumor samples were defined as malignant tumor-conservative TFs including Fral, KLF5, and NFY, while TFs that appeared only once were defined as specific TFs such as POU2F2. Finally, eight common malignant tumor cell lines including 76NF2V and 786-M1A were selected to predict CRC TFs with CRCmapper software. A total of 60 CRC TFs were obtained, among which SMAD3 were present in five types of common malignant tumors, while 46 CRC TFs including NR5A1 were only present in one type of malignant tumor. Taken together, our study provides new ideas for the research of these malignant tumors and experimental validations in cancer cells. However, identified conservative and specific SEs and TFs need to be verified by basic experiments, which might indicate a promising method to improve malignant tumor therapy.

## Appendix

**Table S1:** Primer sequences for ChIP-PCR

Gene	Sequence
SMAD3 (human)	Forward: 5ʹ-CTCCTGTCTTGCCCCACTTT-3ʹ
	Reverse: 5ʹ-GGTTGGACTCGCAGCAAGTA-3ʹ
ETS1 (human)	Forward: 5ʹ-GGTCGTGGGAGGGTTGTTAG-3ʹ
	Reverse: 5ʹ-CCGTCTGATTCTCCACGCAT-3ʹ
HOXB2 (human)	Forward: 5ʹ-AGCCTCTTTCGACTCCCTCT-3ʹ
	Reverse: 5ʹ-CGCGGGGAAAGAGTTTAGGT-3ʹ

**Table S2:** Primer sequences for RT-qPCR

Gene	Sequence
SMAD3 (human)	Forward: 5ʹ-TGGACGCAGGTTCTCCAAAC-3ʹ
	Reverse: 5ʹ-CCGGCTCGCAGTAGGTAAC-3ʹ
ETS1 (human)	Forward: 5ʹ-GATAGTTGTGATCGCCTCACC-3ʹ
	Reverse: 5ʹ-GTCCTCTGAGTCGAAGCTGTC-3ʹ
HOXB2 (human)	Forward: 5ʹ-CGCCAGGATTCACCTTTCCTT-3ʹ
	Reverse: 5ʹ-CCCTGTAGGCTAGGGGAGAG-3ʹ
GAPDH (human)	Forward: 5ʹ-GGAGCGACATCCCTCCAAAAT-3ʹ
	Reverse: 5ʹ-GGCTGTTGTCATACTTCTCATGG-3ʹ

**Table S3:** Data information of eight common malignant tumor cell lines in the SRA database

Malignant tumor type	Number of cell lines	Reference
Gastric cancer	6	Baek Su-Jin et al. 2016 Oncotarget
Renal cancer	4	Baek Su-Jin et al. 2016 Oncotarget
Esophageal squamous cell carcinoma	6	Jiang Yuan et al. 2018 Nature communications.
Colorectal cancer	24	Jiang Yan-Yi et al. 2016 Gut
Bladder cancer	3	Cohen Andrea J et al. 2017 Nature communications
Breast cancer	12	Pattison Jillian M et al. 2016 Molecular cancer research
Small cell lung cancer	14	Franco Hector L et al. 2018 Genome research
Liver cancer	2	Huang Yu-Han et al. 2018 Genes & development

**Table S4:** Information and classification of SE-regulated genes in eight malignant tumor cell lines

SE-regulated target gene type	Gene
Conservative target genes	ABALON, BCL9L, DLGAP1-AS2, LINC00963, EPHA2, MALAT1, MIR21, NEAT1, TNRC18, UBC, etc. (2154 genes in total)
Specific target genes	A2M, AADACL2-AS1, ABCA1, ABCA13, ABCB1, ABCB11, BCC4, ABCG1, ABHD16B, ABHD17C, etc. (35 genes in total)

**Table S5:** Core transcription factors in eight malignant tumor cell lines

Malignant tumor cell line	Transcription factors with the highest score
Breast cancer 76NF2V	SMAD3, RUNX1, KLF5, TCF7L2, SOX9, MAF, ELF3, ERG, KLF4, EHF, FOXC1, MYC, FOSL2, IKZF2, TGIF1, DLX2, FOXD2
Renal cancer 786-M1A	SMAD3, NR3C1, FOXL1, GLIS3, RUNX2, KLF7, NFIL3, ETV6, ETS1, HNF1B, GLI2, KLF4, RUNX1
Small cell lung cancer COR-L311	NFATC1, SOX9, GFI1B, ASCL2, POU2F3, KLF13, PRDM1, INSM1, HMX2, SOX13, MEF2D
Colorectal cancer HCT116	HIF1A, NR5A2, RREB1, JUNB, BHLHE40, HES1, MEF2D, MYC, NR4A1, SMAD3
Liver cancer HepG2	TBX2, MAX, E2F6, RREB1, SMAD3, EHF, SREBF1, ELF1, HES1, FOXA1, FOXQ1, HNF1A, NR5A1, KLF16, FOXP1
Esophageal squamous cell carcinoma KYSE14	SOX2, EOMES, HOXA7
Gastric cancer MKN45	ELF3, RREB1, BHLHE40, JUNB, EHF, HES1, HNF1B, MEF2A
Bladder cancer T24	SMAD3, ETS1, HOXB2
